# Taxifolin, a novel food, attenuates acute alcohol-induced liver injury in mice through regulating the NF-κB-mediated inflammation and PI3K/Akt signalling pathways

**DOI:** 10.1080/13880209.2021.1942504

**Published:** 2021-07-05

**Authors:** Chuanbo Ding, Yingchun Zhao, Xueyan Chen, Yinan Zheng, Wencong Liu, Xinglong Liu

**Affiliations:** a College of Chinese Medicinal Materials, Jilin Agricultural University, Changchun, Jilin, China; b State Local Joint Engineering Research Center of Ginseng Breeding and Application, Changchun, Jilin, China

**Keywords:** Alcoholic liver disease, NF-κB-mediated inflammatory factors, anti-apoptosis, antioxidant

## Abstract

**Context:**

Taxifolin (TAX) has effective anti-inflammatory, antioxidant and hepatoprotective activities, but its potential mechanism has not been revealed.

**Objective:**

To evaluate the potential protective effect of TAX on acute alcohol-induced liver injury in mice.

**Materials and methods:**

Alcoholic liver injury model was established by oral alcohol in mice, and randomly distributed in five groups (*n* = 10): Normal group (oral saline only); Alcohol group (concentration of fermented alcohol: 56%, 6 mL/kg); TAX groups, mice were orally administered with alcohol, and then TAX with doses of 20, 40, 80 mg/kg, respectively. Oral administration was conducted for 6 weeks.

**Results:**

TAX treatment illustrated that the level of alanine aminotransferase (ALT) was reduced to 65.90 ± 2.26 U/L and aspartate aminotransferase (AST) to 33.28 ± 5.62 U/L compared with alcohol group (ALT 124.51 ± 4.40 U/L, AST 61.70 ± 4.09 U/L), while superoxide dismutase (SOD) was increased to 49.81 ± 2.39 U/mg and glutathione (GSH) to 8.16 ± 0.44 μmol/g, but MDA was reversed to 2.53 ± 0.24 nmol/mg. Histopathological examination showed TAX treatment alleviated alcohol-induced hepatocyte necrosis and inflammatory infiltration. Meanwhile, Western blot and rt-PCR indicated TAX reduced IL-6 to 2.49 ± 0.25 pg/mL and TNF-α to 1.79 ± 0.20 pg/mL, and inhibiting NF-κB activation in liver. Moreover, TAX reversed alcohol-induced apoptosis by regulating the expression of PI3K/Akt and its downstream apoptotic factors.

**Conclusions:**

The research provides novel evidence of the hepatoprotective effect of TAX on alcohol-induced liver injury, while also providing the possibility for future treatment of alcoholic liver disease.

## Introduction

Regular heavy drinking is harmful to health, and alcohol affects various body systems. Although its harmful effects vary with individual differences, long-term heavy drinking can lead to many chronic diseases and other serious health problems, which has become a serious public health problem. When the body’s intake of alcohol exceeds the metabolic rate, the excess will accumulate in the blood, leading to changes in normal body functions, and even a binge drinking can cause obvious body damage. Most acute alcoholic liver injury refers to toxic pathological damage to the liver caused by short-term heavy drinking; its incidence and the mortality rate are increasing year by year, and the research has attracted increasing attention (Yang et al. 2020). Alcoholic liver disease (ALD) may develop from hepatic steatosis to alcoholic hepatitis without intervention, and eventually lead to liver fibrosis, alcoholic cirrhosis and even liver cancer (Baghy et al. 2012). However, effective treatments can reverse the symptoms of early alcoholic liver toxicity, so finding effective treatment options is essential.

The liver is an important part of the body’s metabolic system, which can remove many harmful substances from the body, but it is also attacked and damaged by many harmful substances. When the body is stimulated by a large amount of alcohol, about 90% of the alcohol content is metabolized in the liver, which will cause severe hepatotoxicity. Alcohol dehydrogenase in the cytoplasm of liver cells will metabolize ethanol into acetaldehyde, aldehyde dehydrogenase (ALDH) or other isoenzymes. The lack of acetaldehyde dehydrogenase leads to the accumulation of acetaldehyde, and excess acetaldehyde produces a large amount of reactive oxygen species (ROS), which in turn leads to oxidative stress, hepatic stellate cells (HSCs) and cause severe hepatotoxicity (Cui et al. 2019). In addition, the hepatocytes were directly stimulated by the ethanol and acetaldehyde accumulated in the liver, which can cause degeneration and necrosis of liver cells, and aggravate hepatocyte apoptosis (Li et al. 2017). Furthermore, studies have shown that oxidative stress injury caused by excessive accumulation of acetaldehyde may promote the excessive release of macrophages and many proinflammatory factors, including TNF-α, IL-1β, nuclear factor-kappa B (NF-κB), nitric oxide synthase (iNOS), and cyclooxygenase-2 (COX-2) (Li et al. 2016). Therefore, inflammatory injury is a key pathological process leading to the development of alcoholic liver toxicity, which also provides many target references for the clinical treatment of alcoholic liver toxicity.

In recent years, the active ingredients in natural medicinal plants have performed well in liver protection. For example, maltol treats CCl_4_-induced acute liver injury by inhibiting apoptosis and inflammatory response (Liu et al. 2018); the mechanism of garlic protective polysaccharide on liver protective activity is detected by inhibiting TNF-α, TGF-β1 and core proteoglycan (Wang et al. 2018); Gentian leaf extract can exert anti-inflammatory activity by inhibiting the translocation of NF-κB transcription activity (Cui et al. 2019); Green tea extract can reduce liver inflammation by regulating Toll-like receptor (TLR) 2/3 (Wang et al. 2017).

With a flavanol compound of molecular formula C_15_H_12_O_7_, taxifolin (TAX) is a natural active ingredient mainly derived from coniferous plants (An et al. 2008). TAX is commonly used as a natural antioxidant additive in the food industry (Wang et al. 2011), and the European Commission, the United States, the United Kingdom and China authorized an extension of use of TAX-rich extract as a novel food. In recent years, more and more studies have proved that TAX has a positive effect on health and has highly effective pharmacological activities of antioxidant, anti-inflammatory, antitumor and antiviral effects (Chu et al. 1992; Devi and Das 1993; Kawaii et al. 1999; Romero et al. 2007; Weidmann 2012). Although TAX is a natural product with good antioxidant and anti-inflammatory effects, until now there has been no discussion about the molecular mechanism of TAX improving alcoholic hepatotoxicity in mice. Based on the broad pharmacological effects of TAX, this study evaluates the protective activity of TAX against alcohol-induced hepatotoxicity and its mechanism by using a certain degree of alcoholic beverage to establish an animal model of acute alcoholic liver injury in mice.

## Materials and methods

### Chemicals and reagents

**Figure 1.**
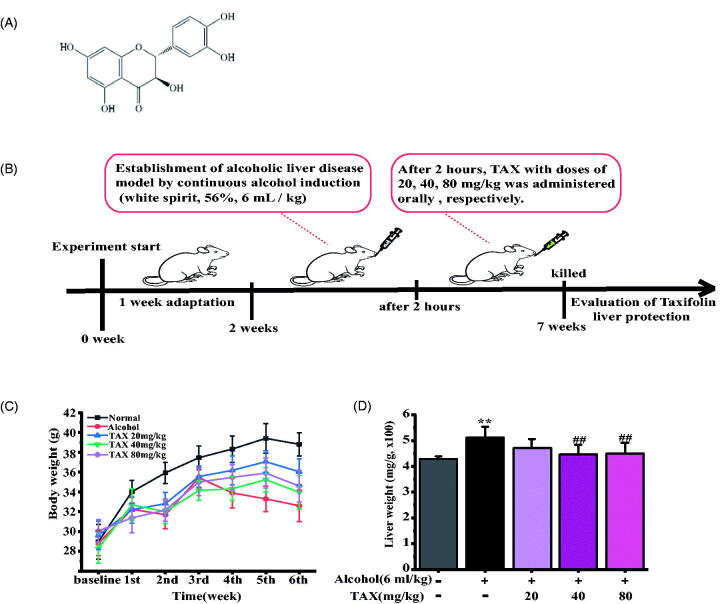
(A) Chemical structure of taxifolin (TAX). (B) The experimental process. (C) Effect of TAX on body weight change among different experimental groups. (D) Liver weight among different experimental groups. Data are mean ± SD. *n* = 10 per group. **p < 0.01, *p < 0.05 vs. Normal group; ##*p* < 0.01, #*p* < 0.05 vs. alcohol group.

The TAX standard was purchased from the National Institutes for Food and Drug Control of China, batch no: 111816-201102, with purity of 98.0%, as shown in Figure 1(A). The Chinese liquor used in the experiment was purchased from Beijing Red Star Co., Ltd. (Concentration of fermented alcohol: 56% vol.; Batch no: 5517042; Beijing, China). Commercial assay kits for aspartate aminotransferase (AST) and alanine aminotransferase (ALT), malondialdehyde (MDA), superoxide dismutase (SOD), glutathione (GSH) assay kit, protein extraction kits, haematoxylin-eosin staining (HE staining) and Masson staining kit were purchased from Nanjing Jiancheng Bioengineering Co., Ltd. (Nanjing, China). The enhanced chemiluminescence (ECL) kit was purchased from Shenyang Wanlei Biotechnology Co., Ltd. (Shenyang, China). Total RNA Rapid Extraction Kit was purchased from Beijing Bioteke Biotechnology Co., Ltd. (Beijing, China); 2 × SYBR Real-Time PCR (rt-PCR) Premix was purchased from TaKaRa Biotechnology Co., Ltd. (Dalian, China). The antibody of rabbit monoclonal anti-mouse inducible iNOS, cyclooxygenase-2 (COX-2), Cytochrome P450 E1(CYP2E1), caspase3 and HRP-conjugated anti-mouse IgG, phosphatidylinositol 3-kinase (PI3K), p-PI3K, protein kinase B (Akt), p-Akt, nuclear factor-kappa B (NF-κB, p65), p-NF-κB (p-p65), inhibitor of κBα (IκBα), p-IκBα, IκB kinase α/β (IKKα/β), p-IKKα/β, B-associated X (Bax), B-cell-lymphoma-2 (Bcl-2), β-actin and secondary antibodies for Western blot were all obtained from Abcam (Cambridge, UK). Other compounds, such as alcohol of different investigative rank, were from Beijing Chemical Factory.

### Animals and experimental plan

The Changchun YISI Experimental Animal Co., Ltd. and the Certificate of Quality is No. SCXK (JI)-2016-0003 (Changchun, China) delivered 50 fully-grown male ICR mice (6–8 weeks old; weight 22–25 g). All animal investigational processes were done according to the Guide for the Attention and usage of Laboratory Animals (Ministry of Science and Technology of China, 2006) and permitted by the Animal Investigational Morals Committee of Jilin Agricultural University, ethics approval number: 2020-12-26-002.

The mice were placed at a precise pathogen-free climate room with ensured sufficient space for movement. Animal care for all mice with litter replacement every day was carried out, while keeping the temperature at 25.0 ± 1.0 °C on 12-h light/dark cycle with free access to food and water. After acclimation for 1 week, mice were randomly distributed in five groups (*n* = 10): Normal group, with mice receiving oral saline only; Alcohol group, mice were orally administered with alcohol (concentration of fermented alcohol: 56%, 6 mL/kg); and three TAX groups, mice were orally administered with alcohol for a period of time to ensure that the alcohol components were completely absorbed, and then TAX with doses of 20, 40, 80 mg/kg, respectively. Oral administration was conducted for 6 weeks.

After 12 h of fasting following the last dose, all mice were anaesthetized by intraperitoneal injection of 70 mg/kg pentobarbital sodium (Shanghai Beizhuo Biochemical & Technological Co., Ltd., Shanghai, China). Serum samples were immediately collected from the eyeballs and placed at room temperature for 40 min, and then separated by centrifugation at 3000 *g* for 10 min at 4 °C for biochemical index analysis. Then, the anaesthetized mice were dislocated to death and immediately dissected, and liver samples were taken. All the dissected mice corpses were delivered to the Experimental Animal Center of Jilin Agricultural University for unified processing. Liver sections were preserved with 10% formaldehyde, and remaining liver segments were preserved at –80 °C for additional experiments.

### Biochemical analysis

By conferring to industrialist’s procedure, and consuming a clinical automatic analyzer (Hitachi, Japan) and a commercial reagent kit (Roche Diagnostic, Mannheim, Germany), levels of ALT, AST were quantified in the serum. Hepatic quantity was quantified conferring to the company’s protocol with hydroxyproline detection kit (Sigma-Aldrich, St. Louis, MO, USA). GSH, SOD, MDA intensities were quantified via commercial kits (lipid peroxidation). According to the producers’ protocols all the processes were accomplished.

### Histological analysis

Liver sections were preserved in 10% formalin, embedded in paraffin and segmented. For histological examination, liver pieces remained tarnished with H&E. By ensuring the standards, degree of liver fibrosis was assessed. For the valuation of collagen constituents in the livers, livers segments were stained with Masson’s trichrome staining. By means of a light microscope and the Quantity One computerized morphometry system (Bio-Rad, Hercules, USA), fibrotic area was measured. Histological valuation was done by randomized assortment.

### Immunohistological and immunofluorescence staining

The paraffin-embedded sections with a thickness of 5 μm were deparaffinized and dehydrated using xylene and ethanol aqueous solution. After antigen retrieval, the liver tissue was washed with phosphate buffer (0.01 M, pH7.4), and then incubated in 1% bovine serum albumin for 1 h. Added mouse polyclonal primary antibodies (1:200), and incubated overnight at 4 °C. Then, it was washed with PBS and incubated with the secondary antibody for 30 min, and then DAB staining and haematoxylin counterstaining were performed. The staining of the sections was observed under a microscope (Olympus, Tokyo, Japan), and the positive expression intensity of the cells was analyzed using Image-Pro Plus 6.0 software.

The expression levels of CYP2E1 and caspase-3 protein in alcohol-induced liver diseases were evaluated by immunofluorescence staining. Preliminary section processing was similar to that of immunohistochemistry, and liver sections were incubated with antibodies CYP2E1 (1:200) and caspase-3 (1:200) at 4 °C for 12 h, and then washed with PBS. Subsequently, the sections were exposed to the secondary antibody (1:400) with dye-488 labelling and kept it at 37 °C for 30 min, and then the nuclei of the fixed tissue were re-stained with 4,6-diamino-2-phenylindole (DAPI). Simultaneously, the degree of immunofluorescence staining was detected by fluorescence microscope (Leica DMILED, Germany), and the positive expression analysis was performed using Image-Pro Plus 6.0 software.

### Western blotting analysis

Using RIPA lysis buffer and phosphatase inhibitor (Sangon Biotech Co., Ltd., Shanghai, China), hepatic tissues were lysed. Thirty micrograms of protein was separated by SDS-polyacrylamide and this was transported on a PVDF membrane after 2 h treatment with bovine serum albumin (BSA). Using primary antibodies [AKT, p-AKT, PI3K, p-PI3K, NF-κB (p65), p-NF-κB, IκBα, p-IκBα, IKK kinase α/β (IKKα/β), p- IKK α/β, Bax and Bcl-2] at 4 °C, the membranes remained incubated for whole night. After incubating with equivalent secondary antibodies (β-actin) proteins intensities were analyzed by Emitter Coupled Logic (ECL) substrate (Pierce Chemical Co., Rockford, IL, USA). Quantity One software (Bio-Rad Laboratories, Hercules, USA) was used for examination of the protein band and analyzed by Quantity One (Bio-Rad) according to standard technique.

### Real-time PCR analysis

Liver sections were process with a TL2020 crushing machine (DHS Life Science & Technology, Beijing, China). Using Trizol reagent (Invirogen), and following the procedure of the manufacturer, whole RNA was removed from the liver sections. The expression intensities of inflammatory factors were examined by using real-time fluorescent quantitative rt-PCR. The reference gene used in this process was β-actin. Using cycle threshold (Ct), the countenance intensities were examined and then using the 2^−ΔΔCt^ method normalized to β-actin appearance. Results were obtained from triplicate experimentations. Table 1 shows the primers used in the PCR reactions.

**Table 1.** Primers sequence.

Genes	Primer forward	Primer reverse
β-actin	TCACTGCCACCCAGAAGAC	GAAGTCGCAGGAGACAACC
IL-6	CCACTTCACAAGTCGGAGGCTTA	CCAGTTTGGTAGCATCCATCATTTC
IL-1β	TCCAGGATGAGGACATGAGCAC	GAACGTCACACACCAGCAGGTTA
TNF-α	TGGCAAATGTGAGAAACGAG	AAACCAGAACAGACCCAACG

### Statistical analysis

All the parameters displayed in the experiment are expressed as mean ± SD, and statistical significance was determined by one-way analysis of variance (ANOVA). SPSS 24.0 software was used to carry out the least significant difference (LSD) multiple comparison test, where *p* < 0.05 or 0.01 were defined as statistically significant difference. The statistical graph was represented by GraphPad Prism 6.0.4 software (GraphPad Software, Inc., San Diego, CA, USA).

## Results

### TAX alleviates alcohol-induced liver injury

Many current studies have proved that TAX has great health benefits, but its protective effect on alcohol-induced hepatotoxicity has not yet been studied (Figure 1(A)). In this study, the hepatoprotective activity of TAX against ALD was evaluated *in vivo*. Taking the dose of 6 mL/kg did not immediately cause the acute death and abnormal performance of mice, so 6 mL/kg/day was used in the following study and the experimental progress is shown in Figure 1(B). Besides, body weight and liver index were included in the evaluation indicators throughout the experiment. The average body weight of normal mice tended to be stable as the development of experiments (36.81 ± 1.18 g), but the average body weight of mice by alcohol-induced displayed a downward trend throughout the experiment (32.60 ± 1.60 g) (*p* < 0.05) (Figure 1(C)), while the weight of all mice treated by TAX was increased (34.59 ± 1.59 g), and liver index (4.703 ± 0.415 mg/g) was significantly lower than that of the alcohol-induced group (5.261 ± 0.255 mg/g) (*p* < 0.01) (Figure 1(D)).

**Figure 2.**
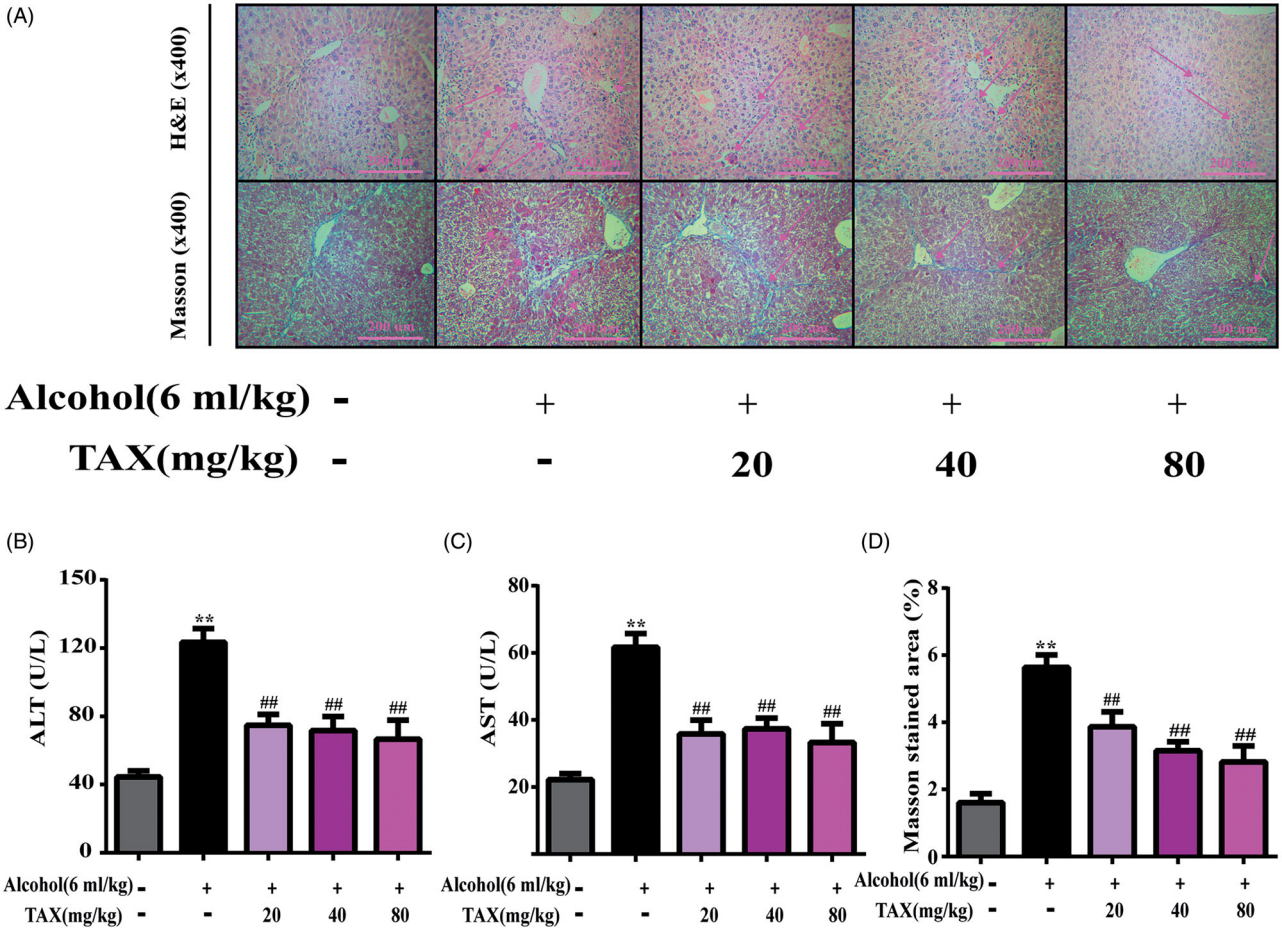
TAX mitigates liver injury and fibrosis in mice. (A) H&E staining of liver tissue and Masson staining of liver tissue, amplification: 400×. Effect of TAX on levels of ALT (B) and AST (C) in serums of mice; (D) Masson relative stained area. Data are mean ± SD. *n* = 10 per group. **p < 0.01, *p < 0.05 vs. normal group; ##*p* < 0.01, #*p* < 0.05 vs. alcohol group.

To assess the effect of TAX on liver function in mice, the levels of two aminotransferases (ALT and AST) in serum were determined. Under alcohol induction, both ALT (124.51 ± 4.40 U/L) and AST (61.70 ± 4.09 U/L) levels were significantly increased (*p* < 0.01), indicating the mouse liver was seriously damaged after a period of continuous alcohol induction (Figure 2(B,C)). However, pre-treatment with TAX (20, 40 and 80 mg/kg) significantly reversed the increase in these two indicators (*p* < 0.01), indicating that TAX effectively improved alcohol-induced liver injury.

### TAX ameliorates liver histopathological changes

Histopathological examination by H&E staining showed (Figure 2(A–D)) that the liver lobules of the normal group were healthy, and the hepatocytes were arranged radially along the central vein. However, there were obvious granular and inflammatory cell infiltrations on the liver surface of the mice in the alcohol group, specifically referring to increased intercellular spaces, severe damage to liver structure, balloon degeneration of hepatocytes and increased fatty vacuoles. Obviously, a large amount of liver cell necrosis and nuclear contraction and loss of function occurred in the alcohol group. In contrast, TAX treatment significantly relieved the hepatocyte structure around blood vessels. Moreover, Masson staining was used to evaluate liver fibrosis incisions, and many fibroblast-like cells and considerable lobular liver injury were also observed in alcohol group. However, the liver pathological changes in the TAX groups were improved, including the decrease of surface fibrosis, regular cell arrangement, the reduction of nuclear degeneration and necrosis.

### TAX treatment ameliorates alcohol-induced oxidative stress injury

**Figure 3.**
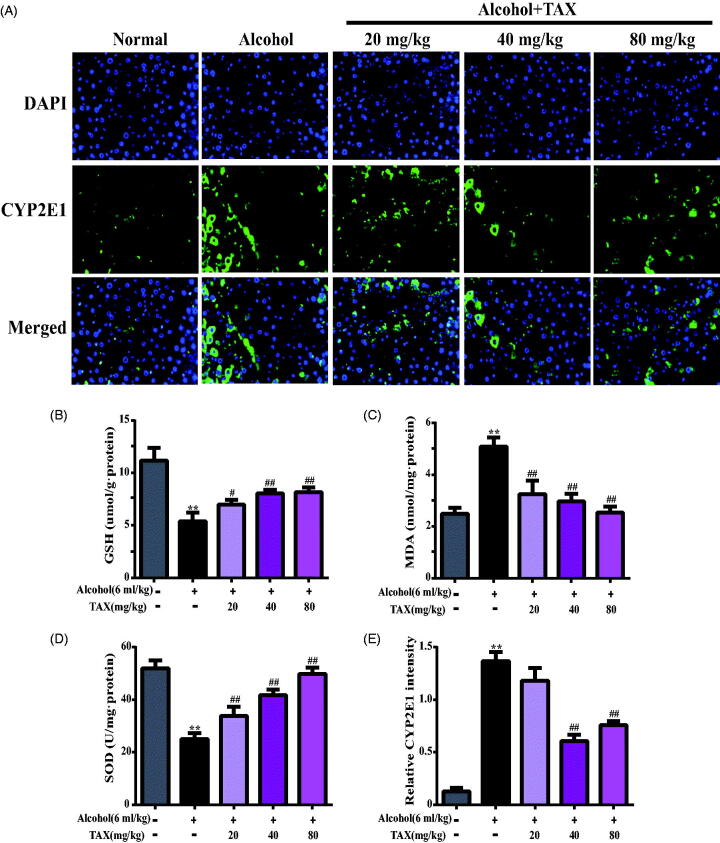
TAX treatment ameliorates alcohol-induced oxidative stress injury. (A) Liver cells stained with immunofluorescence probes of cytochrome P450 E1 (CYP2E1). Effect of TAX on liver GSH (B), MDA (C), and SOD (D) in alcohol-induced mice. (E) Relative CYP2E1 intensity, representative quantification of immunofluorescence images at 200×, 4,6-diamidino-2-phenylindole (DAPI) was used as a nuclear counterstain. Data are mean ± SD. *n* = 10 per group. **p < 0.01, *p < 0.05 vs. normal group; ##*p* < 0.01, #*p* < 0.05 vs. alcohol group.

To further prove whether oxidative stress was involved in alcohol-induced hepatotoxicity in mice, the level of oxidative stress indicators in the liver was examined. Compared with the normal group (GSH: 11.16 ± 1.73 μmol/g; SOD: 51.91 ± 3.06 U/mg), the GSH (5.38 ± 0.43 μmol/g) and SOD (25.05 ± 2.34 U/mg) levels in the liver of the alcohol group were significantly reduced (*p* < 0.01) (Figure 3(B–D)), whereas the MDA (5.08 ± 0.35 nmol/mg) levels were significantly increased (*p* < 0.01) (Figure 3(C)), which indicated that oxidative stress injury by alcohol-induced occurred in the liver of mice. However, MDA (2.53 ± 0.24 nmol/mg) levels in the liver were reversed after treatment with TAX compared with the alcohol-induced group, whereas GSH (8.16 ± 0.44 μmol/g) and SOD (49.81 ± 2.39 U/mg) were significantly increased.

Since CYP-mediated biological activation plays an important role in hepatotoxic diseases, this study examined the protein expression of CYP2E1 after inducing alcohol in the liver. Compared with the normal group, the expression of CYP2E1 metabolic enzymes in the alcohol group was significantly increased (*p* < 0.01) (Figure 3(A–E)). However, TAX pre-treatment effectively reduced the expression of CYP2E1. In summary, TAX reduced the alcohol-induced liver oxidative stress damage to a certain extent.

### TAX treatment inhibits acute alcohol-induced liver inflammatory injury in mice

**Figure 4.**
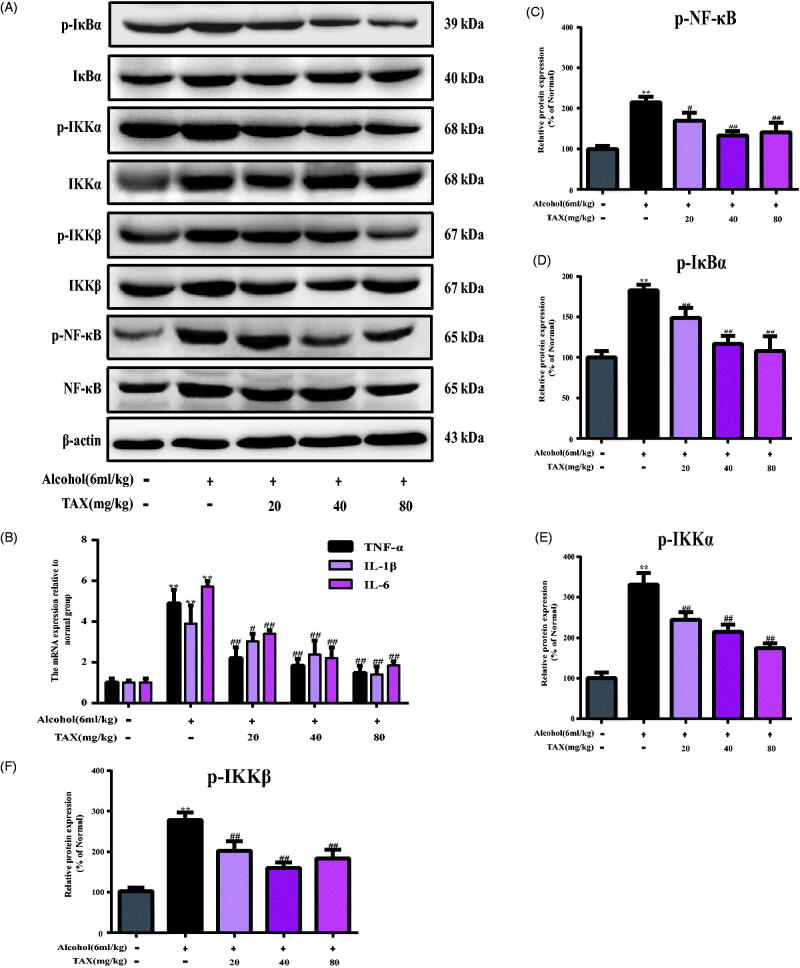
TAX treatment inhibits acute alcohol-induced liver inflammatory injury in mice. (A) Effects of TAX on inflammatory responses. (B) The expression of IL-1β, TNF-α and IL-6 were examined by rt-PCR. The expression of protein p-NF-κB/NF-κB (C), p-IκBα/IκBα (D), p-IKKα/IKKα (E), and p-IKKβ/IKKβ (F) were analyzed by Western blot analysis with specific primary antibodies, and β-actin protein level was used as a loading control. Data are mean ± SD. *n* = 10 per group. **p < 0.01, *p < 0.05 vs. normal group; ##*p* < 0.01, #*p* < 0.05 vs. alcohol group.

**Figure 5.**
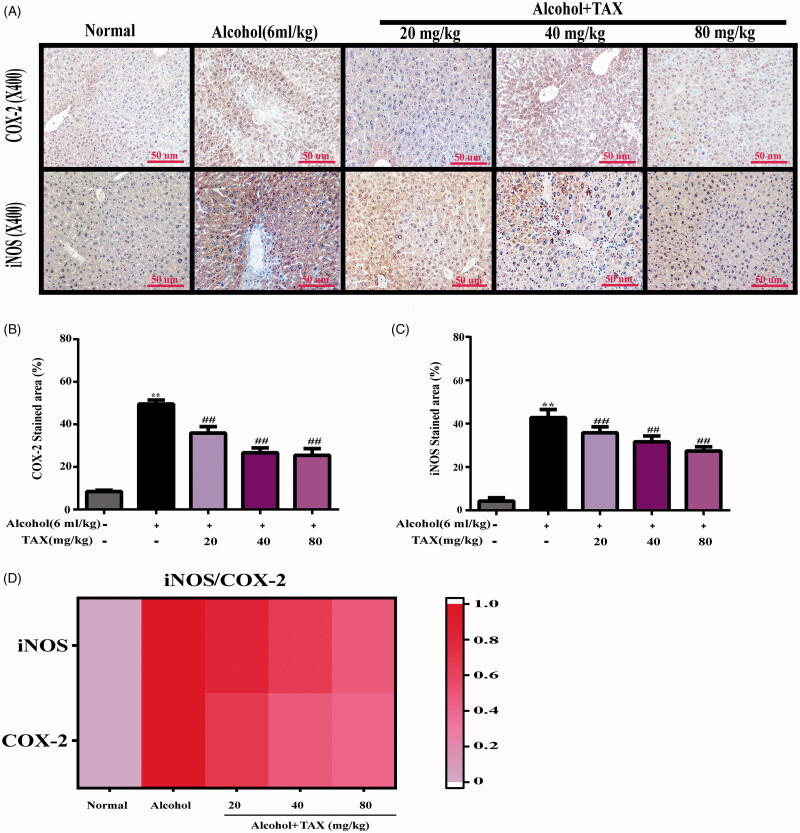
(A) Immunohistochemical staining of COX-2 and iNOS inflammatory factors in liver tissue. (B, C) Inflammatory cell expression area %. (D) Heat map analysis of correlation of iNOS and COX-2 relative stained area. Data are mean ± SD. *n* = 10 per group. **p < 0.01, *p < 0.05 vs. normal group; ##*p* < 0.01, #*p* < 0.05 vs. alcohol group.

In the alcohol-induced liver injury model, inflammatory factors often behave abnormally, and the NF-κB pathway is often used as an important signalling factor in the inflammatory response. Interestingly, the TAX treatment significantly reduced the expression levels of p-NF-κB/NF-κB, p-IκB α/IκB α, p-IKK α/IKK α and p-IKK β/IKK β proteins (*p* < 0.01) (Figure 4(A,C–F)). Meanwhile, it was analyzed by rt-PCR whether TAX could inhibit other inflammatory mediators in alcohol-induced liver tissue. The results showed that the TNF-α (4.23 ± 0.49 pg/mL), IL-1β (3.76 ± 0.14 pg/mL) and IL-6 (4.56 ± 0.45 pg/mL) mRNA levels in the liver of the model group were significantly increased after continuous alcohol-induced compared with the normal group (TNF-α 0.75 ± 0.08 pg/mL; IL-1β 0.61 ± 0.02 pg/mL; IL-6 0.44 ± 0.04 pg/mL), but the mRNA levels of inflammatory factors in each dose group were significantly suppressed after TAX treatment compared with alcohol group, especially the improvement effect of TAX 80 mg/kg was the most obvious (TNF-α 0.79 ± 0.08 pg/mL; IL-1β 1.58 ± 0.06 pg/mL; IL-6 2.49 ± 0.25 pg/mL) (*p* < 0.01) (Figure 4(B)). Moreover, immunohistochemical staining was used to examine the effect of TAX on the expression intensity of iNOS and COX-2 in alcoholic liver tissue. Interestingly, TAX treatment could antagonize overexpression of iNOS and COX-2 after inducing alcohol (*p* < 0.01) (Figure 5(A–C)), and iNOS and COX-2 protein expression levels were more intuitively analyzed through heat map (Figure 5(D)). Indeed, all the above results have effectively confirmed that TAX treatment prevented alcohol-induced inflammation.

### TAX treatment reduces alcohol-induced apoptosis of hepatocytes

**Figure 6.**
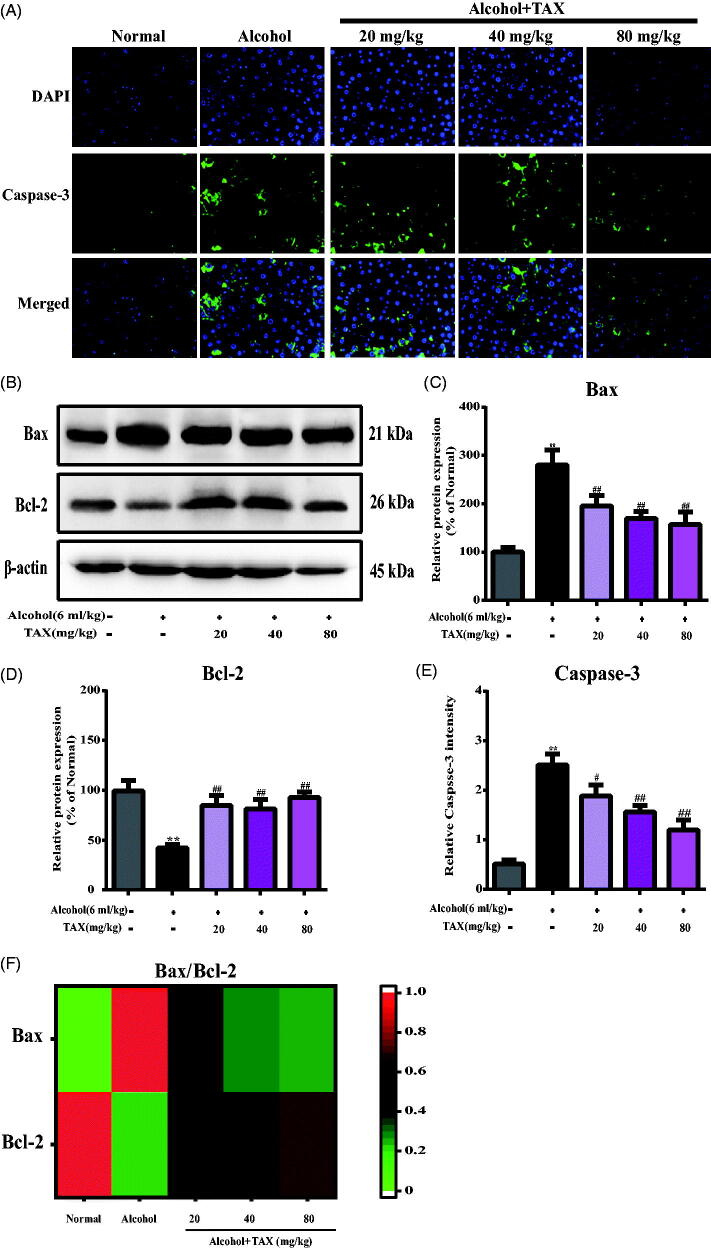
TAX treatment reduces alcohol-induced apoptosis of hepatocytes. (A) Liver cells stained with immunofluorescence probes of Caspase-3. (B) The expression of protein Bax and Bcl-2 were measured, and β-actin protein level was used as a loading control. (C, D) Quantification of relative protein expression was performed by densitometric analysis. (E) Relative Caspase-3 intensity, representative quantification of immunofluorescence images at 200×, 4,6-Diamidino-2-phenylindole (DAPI) was used as a nuclear counterstain. (F) Heat map analysis of of Bax and Bcl-2 protein relative expression levels. Data are mean ± SD. *n* = 10 per group. **p < 0.01, *p < 0.05 vs. normal group; ##*p* < 0.01, #*p* < 0.05 vs. alcohol group.

The Caspase protein family plays an important role in mediating apoptosis. Caspase-3, as a key executive factor, plays a role in many pathways of apoptosis signal transduction. Therefore, to determine whether TAX alleviates alcohol-induced hepatocyte apoptosis, we observed the positive expression of caspase-3 protein by immunofluorescence staining. Almost no abnormally expressed caspase-3 protein factor was observed in the normal group of mice, whereas the expression of caspase-3 in the alcohol group was significantly increased compared with the normal group (*p* < 0.01) (Figure 6(A,E)). Conversely, TAX continuous pre-treatment effectively reduced the expression of caspase-3 (*p* < 0.05).

**Figure 7.**
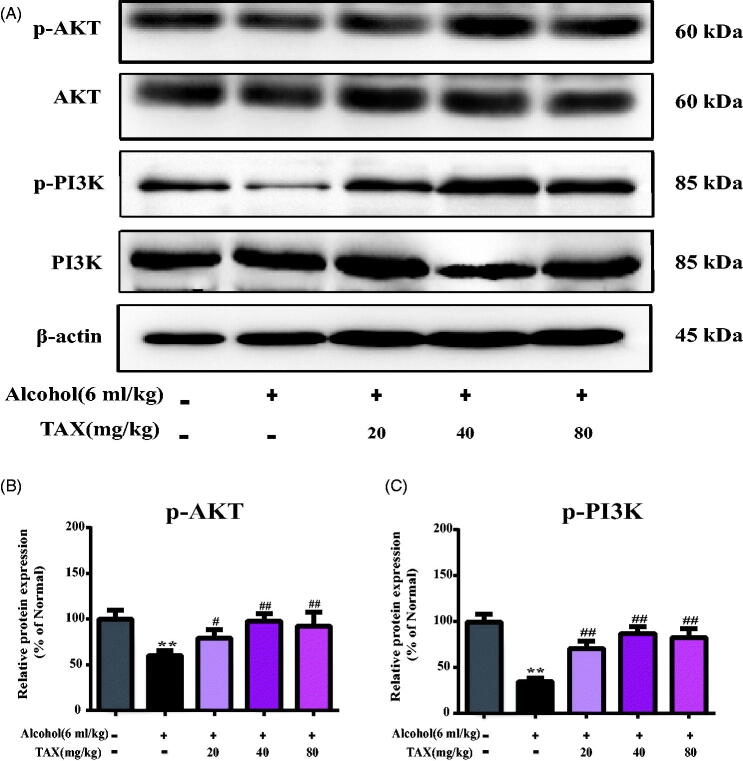
Effects of TAX on PI3K/AKT signalling pathway. (A) The expression of protein p-PI3K, PI3K, p-AKT and AKT were measured, and β-actin protein level was used as a loading control. (B, C) Quantification of relative protein expression was performed by densitometric analysis. Data are mean ± SD. *n* = 10 per group. **p < 0.01, *p < 0.05 vs. normal group; ##*p* < 0.01, #*p* < 0.05 vs. alcohol group.

To further explore the molecular mechanism of TAX against alcohol-induced hepatocyte apoptosis, the PI3K/AKT signalling pathway was analyzed by Western blotting, including its downstream pro-apoptotic factor Bax and anti-apoptotic factor Bcl-2. The results demonstrated that p-PI3K and p-AKT protein levels decreased after continuous alcohol induction, whereas the expression of both was restored after treatment with TAX (*p* < 0.01) (Figure 7(A–C)). In addition, the pro-apoptotic factor Bax was increased significantly whereas the anti-apoptotic factor Bcl-2 decreased (*p* < 0.01) (Figure 6(B–D)) in alcohol-induced liver tissues, and Bax/Bcl-2 protein expression levels were differentially analyzed by heat map (Figure 6(F)). However, TAX (20, 40 and 80 mg/kg) doses significantly reversed these changes, which the above evidences indicated that TAX had anti-apoptotic effect in alcohol-induced hepatocyte apoptosis.

## Discussion

TAX, as a widely existing natural active substance, has high pharmacological activity and therapeutic effect with antioxidant, anti-inflammatory, antitumor and antiviral effects due to its special structure and properties. Therefore, we evaluated the hepatoprotective effect and mechanism of TAX on alcohol-induced liver toxicity in mice (Kandaswami et al. 1992; Sugihara et al. 1999). In this study, the levels of ALT and AST in the serum of the alcohol-treated group were significantly higher than those of the normal group after 6 weeks of modelling, and TAX inhibited the abnormal changes of these two aminotransferases. Similarly, histopathological examination also showed that TAX reduced the infiltration of inflammatory cells in the liver, restored the damaged liver structure to the normal liver lobular structure and reduced collagen deposition.

Recent studies have shown that antioxidant enzymes were the key defense lines for scavenging free radicals and oxidative stress injury in the liver, such as GSH-Px and SOD. The main causes of ALD was oxidative stress injury and the decrease of free radical scavenging activity by alcohol-induced, which has been confirmed in many animal experiments (Han et al. 2015). Concurrently, CYP2E1 is an important drug metabolizing enzyme, and its high activity promotes the production of ROS and leads to the destruction of cell membrane, which seriously affects the function of DNA and protein (Rukkumani et al. 2004; Hau et al. 2009). Once the antioxidant activity of liver is inactivated by ROS and free radicals, the liver will be attacked (Wong et al. 2001), and then the liver will be damaged to produce diseases such as hepatitis, fibrosis and even cirrhosis (Noh et al. 2011). In this study, GSH and SOD activities were enhanced after treatment with TAX in alcohol-induced, and CYP2E1 overexpression was inhibited, which confirmed that TAX reversed alcohol-induced liver toxicity by reducing oxidative stress injury.

In addition to oxidative stress, another important pathogenesis of ALD is inflammation. As a complex physiological and pathological phenomenon, inflammatory injury involves the interaction between multiple cells and molecules. Therefore, it is necessary to strictly and carefully prevent and control inflammation in order to avoid the occurrence of disease (Balkwill et al. 2005; Zhang et al. 2016). The NF-κB signalling pathway is activated in the tissues under the continuous attack of alcohol, whereas activated NF-κB will rapidly phosphorylate, transfer and degrade, and further lead to the release of NF-κB subunits (Hayden and Ghosh 2008). Simultaneously, activated NF-κB stimulates the expression of inflammation-related genes and the release of proinflammatory factors, such as TNF-α, IL-6, IL-1β, iNOS and COX-2, especially iNOS and COX-2 are the key enzymes involved in inflammation (Zhang and Ghosh 2001; Chung et al. 2007), and further enhancing the inflammatory signal (Cildir et al. 2016). In this study, Western blot analysis showed that the expression of the NF-κB pathway and related protein factors were abnormal in the liver of alcohol group mice, and the expression of proinflammatory genes including COX-2 and iNOS were also significantly increased. However, TAX treatment significantly inhibited the activation of NF-κB, and also reduced the expression of p-IκBα, p-IKKα, p-IKKβ, COX-2 and iNOS. In addition, rt-PCR analysis also showed that TAX significantly inhibited the overexpression of TNF-α, IL-1β and IL-6 mRNA.

The overexpression of many proinflammatory factors and the aggravation of inflammatory response will eventually lead to hepatocyte necrosis and apoptosis. Therefore, to further explore the molecular mechanism of TAX improving alcoholic liver toxicity, immunofluorescence staining and Western blot analysis were used to explore the expression of related factors. Phosphatidylinositol 3-kinase (PI3K) signal was involved in the regulation of proliferation, differentiation, apoptosis and glucose transport (Zhou et al. 2018). In recent years, it has been found that PI3K and its downstream protein kinase (Akt) signal pathway were closely related to the occurrence of many human diseases (Daskalopoulos et al. 2016). PI3K interacts with growth factor receptor or connexin with phosphorylated tyrosine residue, causing dimer conformation change and activation, and activated PI3K produces a second messenger PIP3 on the plasma membrane, which binds to the signal protein Akt in the cell and promotes Akt activation (Steelman et al. 2016). Importantly, Akt is a downstream effector of PI3K, and then activated Akt regulates the expression of multiple downstream target proteins, including Bax, Bcl-2, caspase-3/caspase-9, NF-κB, and regulates cell proliferation, differentiation, apoptosis and migration. In this study, TAX increased the expression of p-PI3K and p-Akt, and significantly inhibited Bax and enhanced the expression of Bcl-2 through the PI3K/Akt signalling pathway. Meanwhile, immunofluorescence indicated that the expression of caspase-3 in liver tissue was significantly decreased after TAX treatment. All of this evidence strongly indicates that TAX inhibited apoptosis by regulating the PI3K/Akt signalling pathway.

## Conclusions

**Figure 8.**
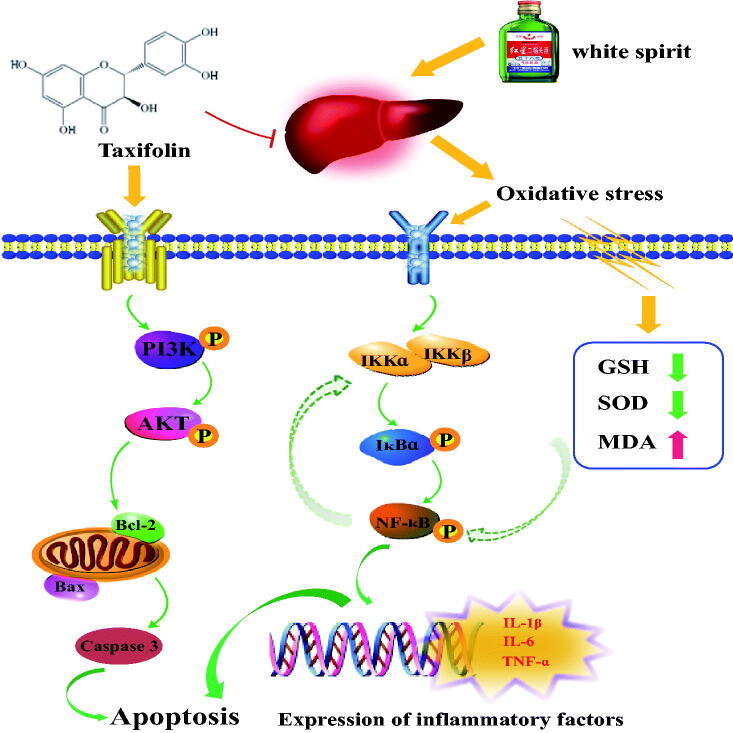
The research summarized TAX the improvement of alcoholic liver disease by activating PI3K/Akt signalling pathway to inhibit apoptosis and inhibiting NF-κB-mediated inflammatory response.

TAX treatment reverses acute alcohol-induced liver injury by alleviating oxidative stress injury and NF-κB-mediated inflammatory response, while improving the PI3K/Akt-mediated apoptosis signalling pathway (Figure 8). Our study demonstrates, for the first time, that TAX treatment can reduce alcoholic liver toxicity, and also provides a reference for future research and treatment of ALD.
